# Mailed faecal immunochemical test programmes and educational inequalities in colorectal cancer screening in Europe: a difference-in-differences analysis

**DOI:** 10.1093/ije/dyag222

**Published:** 2026-10-01

**Authors:** Vladimir Jolidon, Frerik Smit, Katrijn Delaruelle, Cornelia Wagner, Bernadette W A van der Linden, Piet Bracke, Sorana Toma, Claudine Burton-Jeangros, Stéphane Cullati

**Affiliations:** Population Health Laboratory (#PopHealthLab), University of Fribourg, Fribourg, Switzerland; Department of Social and Behavioral Sciences, Harvard T.H. Chan School of Public Health, Boston, MA, United States; Department of Sociological Studies, University of Sheffield, Sheffield, United Kingdom; Institute of Sociological Research, University of Geneva, Geneva, Switzerland; Swiss School of Public Health (SSPH+), Zurich, Switzerland; Swiss Centre of Expertise in Life Course Research (Centre LIVES), Lausanne, Switzerland; Population Health Laboratory (#PopHealthLab), University of Fribourg, Fribourg, Switzerland; Swiss School of Public Health (SSPH+), Zurich, Switzerland; Department of Sociology, Ghent University, Ghent, Belgium; Population Health Laboratory (#PopHealthLab), University of Fribourg, Fribourg, Switzerland; Swiss School of Public Health (SSPH+), Zurich, Switzerland; Swiss Centre of Expertise in Life Course Research (Centre LIVES), Lausanne, Switzerland; Population Health Laboratory (#PopHealthLab), University of Fribourg, Fribourg, Switzerland; Swiss School of Public Health (SSPH+), Zurich, Switzerland; Swiss Centre of Expertise in Life Course Research (Centre LIVES), Lausanne, Switzerland; Department of Sociology, Ghent University, Ghent, Belgium; Equity in (Primary) Health Care Unit, Department of Public Health and Primary Care, Ghent University, Ghent, Belgium; Institute of Sociological Research, University of Geneva, Geneva, Switzerland; Swiss School of Public Health (SSPH+), Zurich, Switzerland; Swiss Centre of Expertise in Life Course Research (Centre LIVES), Lausanne, Switzerland; Population Health Laboratory (#PopHealthLab), University of Fribourg, Fribourg, Switzerland; Institute of Sociological Research, University of Geneva, Geneva, Switzerland; Swiss School of Public Health (SSPH+), Zurich, Switzerland; Swiss Centre of Expertise in Life Course Research (Centre LIVES), Lausanne, Switzerland

**Keywords:** colorectal neoplasms, faecal occult blood test, healthcare disparities, educational status, preventive health services, diagnostic screening programs

## Abstract

**Background:**

Colorectal cancer (CRC) screening has contributed to reducing CRC incidence and mortality in Europe. However, inequalities in screening persist, with lower participation among individuals with lower educational attainment. It remains unclear whether screening programmes can mitigate these inequalities. This study examined the impact of mailed faecal immunochemical test (FIT) programmes on screening participation and related educational inequalities.

**Methods:**

We analysed repeated cross-sectional data from 202 976 respondents aged 50–74 in 24 European countries from the European Health Interview Survey (2014 and 2019), a nationally administered population-based survey. Outcomes were self-reported stool-based screening uptake (lifetime and past 2 years). Educational attainment was categorized into three levels (lower, middle, higher) based on ISCED-2011. We used a difference-in-differences design to compare changes in screening uptake between 2014 and 2019 in countries that implemented nationwide FIT programmes and countries that did not.

**Results:**

In countries that implemented FIT programmes, lifetime and past-2-year screening uptake increased by 28.3 (95% CI: 12.3–44.3) and 30.2 (95% CI: 13.8–46.6) percentage points (ppts), respectively, compared with countries without programmes. The increase was larger among individuals with middle than higher education, with an additional 8.9 (95% CI: 3.3–14.5) ppts for lifetime and 6.4 (95% CI: 1.5–11.2) ppts for past-2-year uptake, indicating a reduction in inequality between these groups. No reduction in inequality was observed between individuals with lower and higher education.

**Conclusion:**

Mailed FIT programmes increased stool-based screening uptake and reduced inequalities between individuals with middle and higher education. To further promote equity in CRC screening, future initiatives should prioritize individuals with lower educational attainment.

Key Messages

- We examined the impact of mailed FIT programmes on screening uptake and educational inequalities across 24 European countries using a difference-in-differences design.
- FIT programmes increased uptake and reduced inequalities between individuals with middle and higher education, but not between those with lower and higher education.
- Equity-oriented strategies are needed to complement FIT programmes and to reach individuals with the lowest educational attainment, ensuring equitable cancer prevention.

## Introduction

Colorectal cancer (CRC) is the third most commonly diagnosed cancer and the second leading cause of cancer-related mortality worldwide [1, 2]. In Europe, the burden remains high, with an estimated 594 000 new CRC cases and 284 000 deaths in 2021, corresponding to age-standardized incidence and mortality rates of 37.3 and 16.9 per 100 000, respectively [2, 3]. CRC is preventable through screening, as stool-based tests and endoscopy reduce both incidence and mortality [4–6]. Notably, European countries with large-scale screening programmes have shown greater declines in CRC incidence and mortality than those without such programmes [7].

The Council of the European Union’s 2022 recommendation advises that CRC screening be organized and population-based, with systematic invitations to individuals aged 50–74, using faecal immunochemical test (FIT) as the preferred method [8]. However, CRC screening strategies across Europe remain heterogeneous: while some countries have adopted organized approaches, others rely on opportunistic screening–offered by healthcare providers, during medical visits, or initiated by individuals–without a centralized invitation system [9, 10].

Several European countries have introduced screening programmes that mail FIT kits directly to individuals, allowing self-administration and return by post. This approach improves accessibility and increases participation [11]. Two recent European studies reported higher CRC screening coverage in countries with nationwide programmes compared with those without [9, 12]. Nevertheless, causal evidence on the impact of nationwide FIT programmes on screening participation in Europe remains limited.

In parallel, research has consistently documented educational inequalities in cancer screening, with individuals of lower educational attainment reporting lower participation across different tests [13–15]. Moreover, cross-national studies in Europe have found that educational inequalities are smaller in countries with organized screening programmes compared with those relying on opportunistic screening [16–18]. These studies suggest that organized programmes may reduce screening inequalities by improving access through systematic invitations to all eligible individuals, but they have not applied causal frameworks, such as quasi-experimental designs.

Regarding CRC screening specifically, evidence on whether FIT programmes reduce screening inequalities remains limited. Two randomized trials found that mailed FIT interventions among patients from clinics and healthcare centres in the United States did not reduce socioeconomic inequalities in participation [19, 20]. However, neither study examined educational inequalities. A Canadian study assessed educational inequalities but reported no evidence that FIT programmes reduced them [21]. Notably, these North American studies did not evaluate nationwide FIT programmes and their findings have limited transportability to the European context. In sum, the impact of FIT programmes on educational inequalities in screening participation remains understudied in Europe.

Between 2014 and 2019, four European countries implemented nationwide CRC screening programmes using mailed FIT kits. Leveraging this quasi-experimental context, we examined the causal effect of these programmes on stool-based screening participation and related educational inequalities.

## Materials and methods

### Data

We used anonymized individual-level microdata from the second (EHIS-2) and third (EHIS-3) waves of the European Health Interview Survey (EHIS). The EHIS is a nationally administered, population-based, repeated cross-sectional survey using probabilistic sampling that collects information on health status, health determinants, and healthcare use among individuals aged 15 and older living in private households [22]. EHIS-2 was conducted between 2013 and 2015 in 28 European Union Member States, plus Iceland and Norway, and EHIS-3 took place between 2018 and 2020 in the same countries.

EHIS data are collected through face-to-face interviews, telephone interviews, or self-administered questionnaires [22]. Our analytical sample included 24 countries that participated in both the EHIS-2 and EHIS-3, and met the requirements of our difference-in-differences study design, as detailed in the next section. The sample was restricted to individuals aged 50–74, the age group eligible for CRC screening according to European guidelines [10]. In Belgium, Luxembourg, and the Netherlands, the sample was restricted to individuals aged 55–74, in line with their FIT programmes’ target population. After excluding cases with missing information on predictors (*n* = 913, 0.43%) and the outcome (*n* = 6772, 3.22%), the final analytical sample consisted of 202 976 respondents.

### Measures

#### Outcome measures

EHIS respondents were asked when they last underwent a faecal occult blood test, with response options including ‘within the past 12 months’, ‘1 to less than 2 years ago’, ‘2 to less than 3 years ago’, ‘more than 3 years ago’, or ‘never’. If necessary, interviewers could explain that the test aims ‘to detect subtle blood loss in the gastrointestinal tract, anywhere from the mouth to the colon’ [22]. We examined two self-reported outcomes of stool-based screening uptake: lifetime uptake (ever versus never) and past-2-year uptake (within the past 2 years versus more than 2 years ago or never). This 2-year interval aligns with the European cancer screening recommendations [10].

### Country-level intervention: mailed FIT programmes

The country-level intervention was the implementation of CRC screening programmes that mailed FIT kits to all eligible individuals. Between EHIS-2 and EHIS-3, four European countries (Belgium, Denmark, Luxembourg, and the Netherlands) introduced such programmes nationwide. We therefore classified countries into two groups: those that implemented mailed FIT programmes during this period and those that did not (Supplementary Table 1). Classification was based on published literature, national reports, and contacted screening programme stakeholders [9, 10, 23–26].

In summary, respondents from Belgium, Denmark, Luxembourg, and the Netherlands (*n* = 16 942) were classified as exposed to mailed FIT programmes between 2014 and 2019 (intervention group), while respondents from 20 other European countries (*n* = 186 034), where no nationwide FIT programmes were implemented during or before this period, were assigned to the comparison group (Supplementary Table 1). Countries were included based on data availability at two time points (before and after intervention). EHIS-3 microdata for France and the United Kingdom were not available to researchers and these countries were therefore excluded. Slovenia, Ireland, and Croatia were excluded because FIT programmes were introduced before the intervention period. Czechia was also excluded as its nationwide programme involved invitation letters only, without mailing FIT kits.

### Individual-level inequality measure: educational attainment

Educational attainment was used to measure inequality in screening uptake. In the EHIS, respondents reported their highest level of education completed, which was recorded following the 2011 International Standard Classification of Education (ISCED-2011; levels 0–8). We recoded the education variable into three categories: (0) higher (ISCED 5–8; tertiary education, including bachelor, master, and doctoral levels); (1) middle (ISCED 3–4; upper secondary and post-secondary non-tertiary); and (2) lower (ISCED 0–2; pre-primary, primary and lower secondary education).

### Statistical analysis

We first calculated descriptive statistics for our analytical sample. Subsequently, we applied a difference-in-differences (DiD) model to assess the causal effect of mailed FIT programmes (the intervention) on CRC screening uptake and educational inequalities in uptake.

Model 1 included the intervention variable (0 = comparison group; 1 = intervention group), the post-intervention period variable (0 = pre-intervention; 1 = post-intervention) and the interaction term *DiD = Intervention × Post*. The interaction term equals 1 for respondents in an intervention country during the post-intervention period. The model also included country dummies (for all but one reference category country) to account for unobserved country-level heterogeneity. The estimation equation is as follows:


([1]) $$Y_{i}=\beta_{0}+{\beta_{1}I\mathrm{ntervention}}_{i}+{\beta_{2}P\mathrm{ost}}_{i}+\beta_{3}{\mathrm{DiD}}_{i}+{{\sum_{c}\beta }_{4}C\mathrm{ountry}}_{c}+\varepsilon_{i}\;$$

$\beta_{1}$ represents differences in screening uptake between intervention and comparison groups prior to the intervention, while $\beta_{2}$ reflects changes in screening uptake over time in the comparison group, accounting for temporal trends unrelated to the intervention. The parameter of interest, $\beta_{3}$, is the average treatment effect of FIT programmes on Y, the uptake of stool-based screening. Given our binary dependent variables, we followed Angrist and Pischke [27] and estimated linear probability models with clustered standard errors at the country level (i.e. the level where the intervention is assigned).

Model 2 was specified with additional variables and interaction terms between education level and the intervention and post-intervention variables to capture the heterogeneous treatment effect (HTE) across education levels:


([2]) $$Y_{i}=\beta_{0}+{\beta_{1}I\mathrm{ntervention}}_{i}+{\beta_{2}P\mathrm{ost}}_{i}+{\beta_{3}DiD}_{i}+{{\sum_{c}\beta }_{4}C\mathrm{ountry}}_{c}+{\beta_{5}E\mathrm{ducation}}_{i}+{\beta_{6}Age}_{i}+{\beta_{7}Sex}_{i}+{\beta_{8}(\mathrm{Intervention}\times \mathrm{Education})}_{i}+{\beta_{9}(\mathrm{Post}\;\times \;Ed\mathrm{ucation})}_{i}+{\beta_{10}(\mathrm{DiD}\;x\;\mathrm{Education})}_{i}+\varepsilon_{i}\;$$

$\beta_{10}$ captures the HTE, indicating how much the impact of the programmes differs by educational level. The model includes important confounders identified in the literature: age and sex, and country fixed effects. To prevent bias in causal estimation, we avoid adjusting for mediating variables [28].

Figure 1 summarizes the study aims, illustrating both the impact of FIT programmes on stool-based screening uptake and their moderating effect on educational inequalities in uptake. Model assumptions and robustness checks were reported in Supplementary Material (see Appendices B and C). Analyses were performed using StataSE 18.

**Figure 1:**
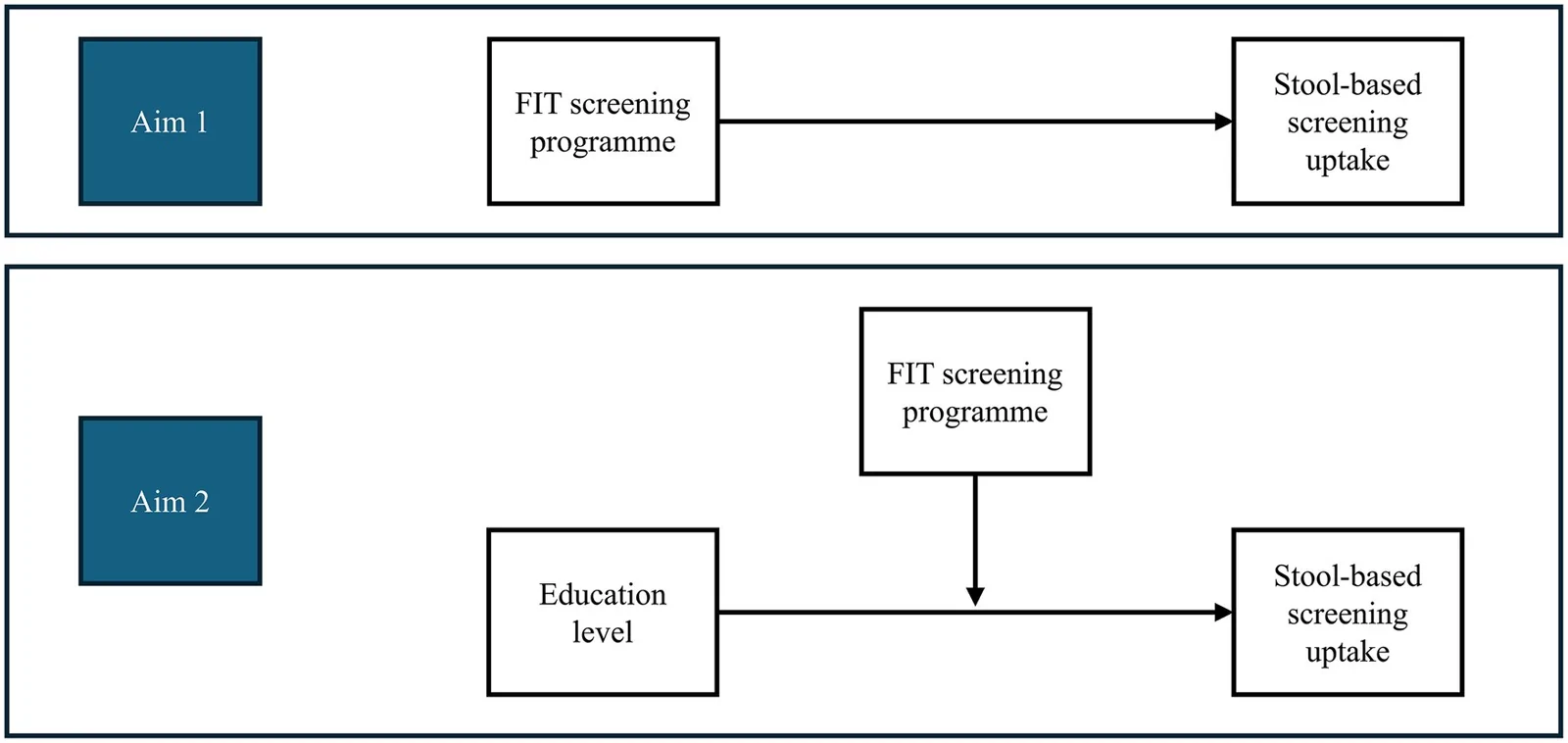
Study aims: effect of FIT programmes on stool-based screening uptake and on educational inequalities in uptake.

## Results

Table 1 presents the descriptive statistics of our analytical sample. Overall, 42.1% of respondents reported undergoing a stool-based test at least once in their lifetime and 26.3% in the past 2 years. A slight majority of respondents were female (54.3%). Regarding educational attainment, 34.9% of participants had primary or lower secondary education (lower), 42.0% had achieved upper secondary education (middle), and 23.1% had tertiary education (higher).

**Table 1:** Sample descriptive statistics, EHIS-2 and EHIS-3 (*n* = 202 976).^a^

	*n*	%
**Stool-based screening in lifetime**		
Never in lifetime	117 549	57.9
Ever in lifetime	85 427	42.1
**Stool-based screening in the past 2 years**		
Never or more than 2 years ago	149 623	73.7
In the past 2 years	53 353	26.3
**Sex**		
Female	110 124	54.3
Male	92 852	45.7
**Education level**		
Lower	70 826	34.9
Middle	85 340	42.0
Higher	46 810	23.1
**Age**		
50–54	40 631	20.0
55–59	43 780	21.6
60–64	43 102	21.2
65–69	41 043	20.2
70–74	34 420	17.0

a *Source:* second and third waves of the European Health Interview Survey (EHIS-2 and EHIS-3).

### Impact of FIT programmes on stool-based screening uptake

Table 2 presents the difference-in-differences (DiD) estimates (i.e. $\beta_{3}$ from Model 1) capturing the impact of FIT programmes on stool-based screening uptake. The introduction of FIT programmes increased the probability of lifetime and past-2-year screening uptake by 28.3 (95% CI: 12.3–44.3) and 30.2 (95% CI: 13.8–46.6) percentage points (ppts), respectively.

**Table 2:** Effect of FIT programmes on stool-based screening uptake, results from difference-in-differences regression models.^a^

	In lifetime			In the past 2 years		
	*b*	95% CI	*P-*value	*b*	95% CI	*P-*value
Intervention (ref.: comparison group)	−0.462	−0.546, −0.379	<.001	−0.302	−0.387, −0.216	<.001
Post (ref.: pre-intervention period)	0.059	0.013, 0.106	.013	0.044	0.002, 0.087	.040
DiD	0.283	0.123, 0.443	.001	0.302	0.138, 0.466	<.001
Intercept	0.767	0.744, 0.789	<.001	0.515	0.494, 0.536	<.001
*R* ^2^	0.23			0.15		
*n*	202 976			202 976		

a DiD (difference-in-differences) denotes the *Intervention* × *Post* term. Models were adjusted for country fixed effects. Standard errors were clustered at the country level. CI, confidence interval.

### Impact of FIT programmes on educational inequalities in stool-based screening uptake

An educational gradient in stool-based screening uptake was observed during the pre-intervention period, in both the intervention and comparison groups, where respondents with higher educational attainment reported higher uptake than those with middle and lower education levels (Fig. 2). These differences across education levels were statistically significant for both lifetime and past-2-year uptake (Supplementary Table 2). In the post-intervention period, stool-based screening uptake within the intervention group was highest among individuals with middle education (71.3%), exceeding the higher (68.2%) and lower (64.5%) education groups (Fig. 2). In the comparison group, individuals with lower and middle education had similar uptake levels (41.5% and 42.6%, respectively), both lower than the screening uptake among individuals with higher education (51.7%).

**Figure 2:**
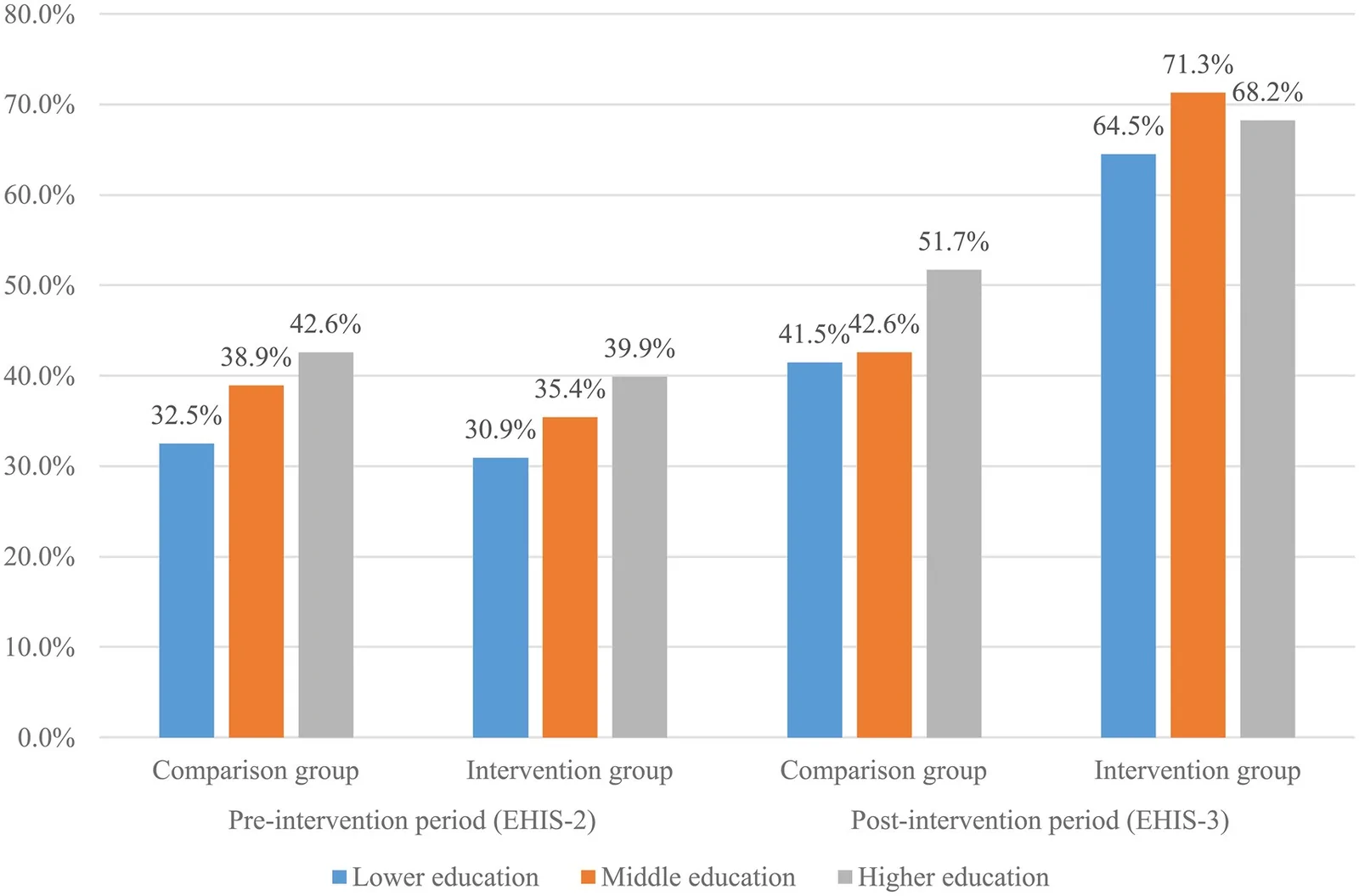
Proportion (%) of respondents who ever had a stool-based screening in their lifetime in intervention and comparison groups, before and after FIT programme implementation, by education level. *Source*: second and third waves of the European Health Interview Survey (EHIS-2 and EHIS-3).

Figure 3 presents the impact of FIT programmes across education levels, as captured by β_3_ and β_10_ in Model 2 (see Supplementary Table 3 for full model specifications including covariates). In this model, the DiD term (*Intervention × Post*) represents the effect of FIT programmes on screening uptake among individuals with higher education (the reference category). In this group, screening uptake increased significantly by 24.6 (95% CI: 9.5–39.7) ppts for lifetime uptake and 27.8 (95% CI: 12.4–43.3) ppts for past-2-year uptake. The three-way interaction shows a significantly greater increase among individuals with middle education relative to the higher education group, by an additional 8.9 (95% CI: 3.3–14.5) ppts for lifetime uptake and 6.4 (95% CI: 1.5–11.2) ppts for past-2-year uptake. This suggests a reduction in screening inequalities between the middle and higher education groups. Among the lower education group, screening uptake increased by 3.5 (95% CI: −6.5 to 13.5) ppts for lifetime uptake and 1.7 (95% CI: −6.9 to 10.3) ppts for past-2-year uptake, relative to the higher education group. However, this increase was not statistically significant, indicating that the programmes did not reduce inequality between the lower and higher education groups.

**Figure 3:**
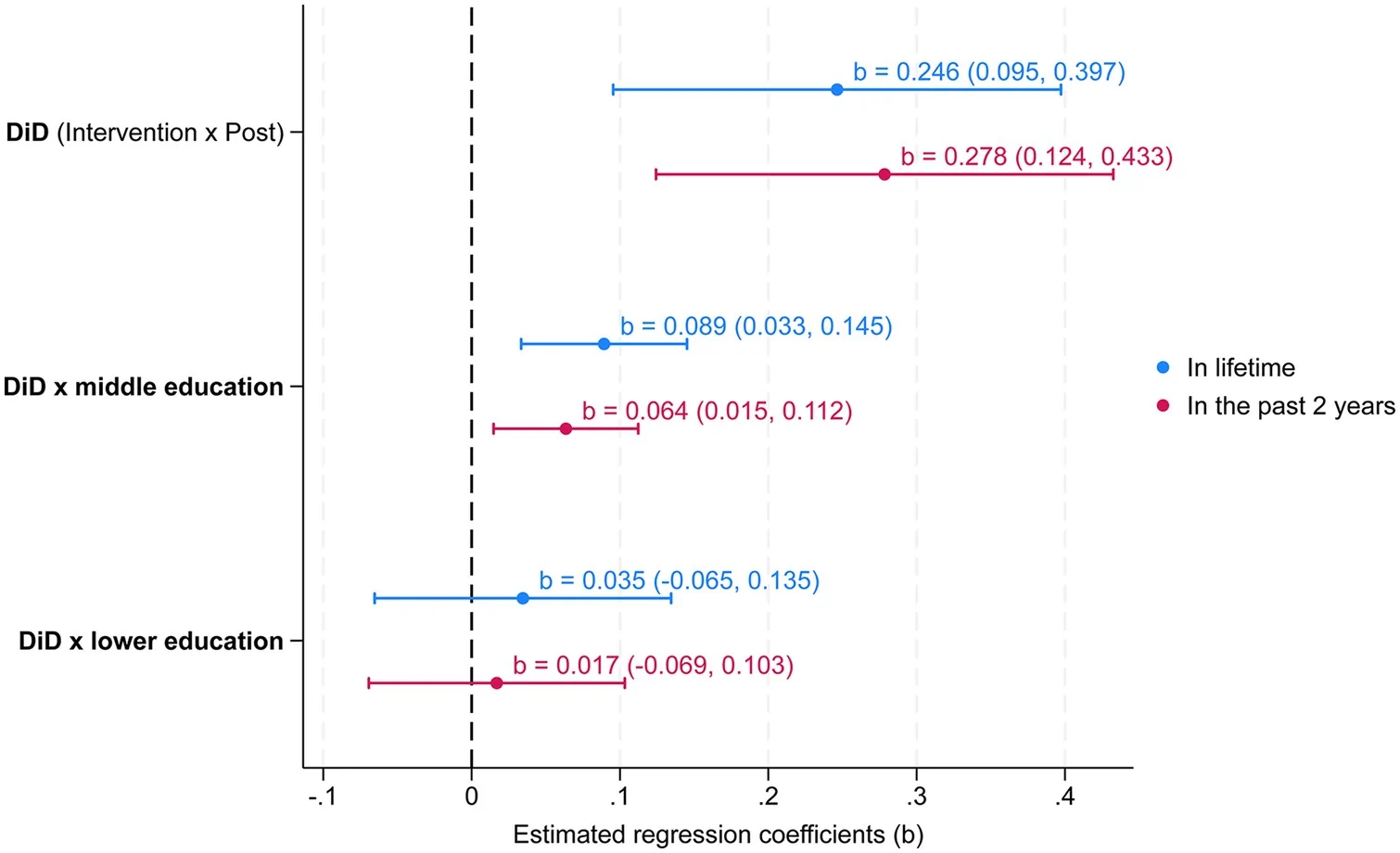
Effect of FIT programmes on stool-based screening across education levels, results from difference-in-differences regression models. *Notes*: bars indicate 95% CIs. DiD (difference-in-differences) denotes the Intervention × Post term. It corresponds to the effect of FIT programmes on screening uptake among the higher education group, i.e. the reference category in the three-way interaction between Intervention ×Post × Education. Models were adjusted for age, sex, and country fixed effects. Standard errors were clustered at the country level.

Causal identification assumptions were assessed and generally supported our analytical strategy (Supplementary Material Appendix B). Pre-intervention trends in Belgium and the comparison countries were consistent with the parallel trends assumption. For other intervention countries, this could not be verified due to limited EHIS data. However, baseline screening uptake was similar between intervention and comparison groups (EHIS-2), supporting the plausibility of parallel trends. Additionally, the intervention was not associated with changes in placebo outcomes, suggesting that the observed effects are unlikely to be driven by broader shifts in healthcare utilization or other time-varying confounders. Falsification tests conducted on non-targeted age groups yielded no significant effects, providing support for the plausibility of our counterfactual comparison.

Finally, robustness checks showed that FIT programmes increased screening uptake in all intervention countries (Supplementary Table 7), with lifetime uptake gains ranging from 11.4 ppts in Luxembourg to 48.4 ppts in the Netherlands. In all four countries, increases were larger among individuals with middle relative to higher education for lifetime uptake, ranging from 4.3 ppts in Denmark to 15.8 ppts in Luxembourg (Supplementary Fig. 2). For past-2-year uptake, inequality reduction was observed in Belgium and Denmark but not in Luxembourg or the Netherlands, making results only partially consistent with the main analysis. Country-specific analyses are, however, subject to reduced statistical power and a higher likelihood of Type II error, which may limit the detection of inequality reduction effects.

## Discussion

This study assessed the impact of organized population-based FIT programmes on stool-based screening uptake and related educational inequalities. Leveraging mailed FIT programme implementation in four European countries between 2014 and 2019, our difference-in-differences analysis showed that these programmes substantially increased screening uptake, by 28.3 ppts for lifetime uptake and 30.2 ppts for past-2-year uptake. Screening inequalities narrowed between individuals with middle and higher education, with uptake increasing by an additional 8.9 ppts (lifetime) and 6.4 ppts (past-2-year) among individuals with middle education compared to those with higher education. Country-specific analyses confirmed this pattern for lifetime uptake in all four intervention countries, and for past-2-year uptake in two countries. Our analyses did not show a significant reduction in screening inequalities between the lower and higher education groups.

While two studies reported higher screening coverage in European countries with nationwide CRC screening programmes [9, 12], our results provide causal evidence quantifying the increase in uptake specifically attributable to mailed FIT programmes. The observed impact is consistent with previous research, including a systematic review reporting a median 21.5% increase in screening uptake from mailed FIT interventions [11]. Our findings further support the effectiveness of direct mailing strategies in improving CRC screening coverage.

Our study makes a key contribution by examining the causal effect of mailed FIT programmes on educational inequalities in screening uptake, providing evidence that these programmes help reduce inequalities between middle and higher education groups. To our knowledge, no previous study has assessed the impact of nationwide programmes on educational inequalities in screening uptake using a quasi-experimental design and population-based data from multiple countries. Previous European studies have suggested that countries with organized screening programmes have smaller screening inequalities than those relying on opportunistic strategies, across different cancer screening tests [16–18]. However, they did not establish causality, and their findings may reflect broader contextual factors rather than the programmes’ effect.

Prior studies have focused on whether FIT programmes increase screening uptake among socioeconomically disadvantaged populations [29], but few have evaluated their impact on inequalities in the general population [21]. Our findings contrast with two randomized trials in the United States, which reported no reduction in socioeconomic inequalities following mailed FIT interventions [19, 20]. These trials were conducted among patients within healthcare systems, included approximately 9700 and 30 700 participants, took place over relatively short follow-up periods (2016–2017 and 2017–2018), and relied on neighbourhood-level socioeconomic indicators. In contrast, our study used individual-level (albeit self-reported) measures of educational attainment and screening uptake in a large population-based sample spanning 24 European countries. Restriction to healthcare users, together with differences in the measurement of inequality (i.e. the use of neighbourhood-level socioeconomic indicators in the US trials), may partly explain the contrasting findings. A Canadian study including approximately 3100–6600 respondents per participating province, with post-intervention periods of 1–4 years, found no reduction in educational inequalities overall, and stratified analyses showed increased inequalities in one province [21]. However, educational attainment in this study was measured using a binary indicator (less than high school versus high school and above), which may capture inequalities differently from the three-category educational measure used in our analysis. Our study contributes to this limited literature by applying a causal design to evaluate the effect of mailed FIT programmes on screening inequalities.

Furthermore, we observed a larger reduction in inequality in lifetime uptake than in past-2-year uptake, between the middle and higher education groups. This finding aligns with previous research suggesting that screening programmes may be more effective in reaching individuals with lower socioeconomic position who have never undergone screening, thereby reducing inequalities in lifetime screening outcome [30].

Nevertheless, we found no clear evidence of inequality reduction between individuals with lower and higher educational attainment. This may reflect enduring barriers, such as lower health literacy, competing life demands and limited engagement with preventive healthcare, that are unlikely to be overcome by mailed FIT kits alone. While such programmes improve overall participation, they may be insufficient to close the gap for individuals with the lowest educational attainment. These findings highlight the need to incorporate equity-focused components into programme design. Systematic reviews have shown that combining mailed outreach with strategies such as enhanced reminders, general practitioner endorsement or patient navigation can further increase uptake among underserved populations [29, 31–33].

Following FIT programme implementation, the educational gradient in screening uptake changed in intervention countries: lifetime uptake was highest among individuals with middle education (71.3%), followed by those with higher (68.2%) and lower education (64.5%). Since Europe’s Beating Cancer Plan set a target of offering CRC screening to 90% of the eligible EU population [8], it is essential to monitor not only overall screening coverage but also educational disparities to ensure equitable progress towards this target and that no subgroup is left behind.

Our study has limitations. Due to limited pre-intervention data, we could assess the parallel trends assumption for only one intervention country, and the results supported the assumption. Although we adjusted for individual-level (age, sex) and country fixed effects, residual confounding cannot be ruled out. Sensitivity analyses using placebo outcomes suggest the estimated impact of the programmes was unlikely due to time-varying confounding, such as secular changes in healthcare utilization. In some intervention countries, the launch of FIT programmes partially overlapped with the EHIS-2 (2014) data collection, possibly resulting in some baseline exposure and underestimation of programme effects. However, early rollout phases had limited coverage, and the gradual implementation makes such exposure likely low. By-country analyses supported the robustness of our results. EHIS is conducted by national statistical agencies based on nationally representative probabilistic samples, harmonized questionnaires and standardized data collection protocols. Data are drawn from population registers, censuses or other statistical and administrative sources and undergo Eurostat validation procedures, thereby supporting validity, reliability and cross-country comparability of the data [22, 34]. Despite a common regulatory framework, national implementation varied across countries, particularly in survey mode, fieldwork period, and translation procedures. Nevertheless, quality reports indicate overall sufficient or good cross-country comparability [22, 35]. Non-response also varied substantially across countries, which may have affected representativeness. In addition, screening participation was self-reported and may therefore be subject to recall or social desirability bias. We did not apply survey weights. While weighting can improve population-level inference, our objective was to estimate effects using a difference-in-differences design. Estimates should therefore be interpreted as applying to the surveyed populations rather than as population-representative estimates. The EHIS did not collect information on whether screening was for preventive or diagnostic purposes, preventing us from specifically assessing the prevalence of asymptomatic preventive screening. Finally, the short pre-post observation timeframe limits our ability to assess the long-term impact of FIT programmes.

## Conclusions

Organized CRC screening programmes with mailed FIT kits, implemented in four European countries, reduced screening inequalities between individuals with middle and higher education levels, especially for lifetime screening uptake as confirmed by country-specific analyses. However, no significant reduction of the gap was observed for the lower education group. These findings highlight the importance of complementing FIT programmes with equity-oriented strategies to ensure that gains in cancer prevention reach all segments of the population.

## Ethics approval

This observational study used anonymized data provided by Eurostat and did not require ethical approval.

## Supplementary Material

dyag222_Supplementary_Data

## Data Availability

Data from the European Health Interview Survey are publicly available at no cost; however, access to anonymized microdata requires a formal application to Eurostat. Information on how to request access can be found at https://ec.europa.eu/eurostat/web/microdata/european-health-interview-survey.
