# Supplementary material for: Mailed faecal immunochemical test programmes and educational inequalities in colorectal cancer screening in Europe: a difference-in-differences analysis
Source: Int J Epidemiol. 2026 Oct 1;55(5):dyag222. doi: 10.1093/ije/dyag222 (PMC13629549; doi:10.1093/ije/dyag222)
Supplement: Supplementary file 1 [file dyag222_supplementary_data.docx]

**Mailed Faecal Immunochemical Test Programmes and Educational Inequalities in Colorectal Cancer Screening in Europe: A Difference-in-Differences Analysis**

Vladimir Jolidon, Frerik Smit, Katrijn Delaruelle, Cornelia Wagner, Bernadette W. A. van der Linden, Piet Bracke, Sorana Toma, Claudine Burton-Jeangros, Stéphane Cullati

**ONLINE SUPPLEMENTARY MATERIALS**

Table of Contents

[Appendix A: Supplementary Tables	2](#_Toc207807149)

[Table S.1 European Health Interview Survey data availability and classification of countries as intervention and comparison groups	2](#_Toc207807150)

[Table S.2 Stool-based screening uptake by education level, before and after FIT programme implementation, in intervention and comparison groups	3](#_Toc207807151)

[Table S.3 Effect of FIT programmes on stool-based screening uptake by education level, results from difference-in-differences regression models	3](#_Toc207807152)

[Appendix B: Model assumptions	4](#_Toc207807153)

[Assessment of the parallel trends assumption	4](#_Toc207807154)

[Figure S.1 Parallel trends assumption in stool-based screening uptake	4](#_Toc207807155)

[Table S.4 Regression-based test of pre-intervention trends in stool-based screening uptake, Belgium (intervention) versus comparison group of countries	5](#_Toc207807156)

[Falsification and placebo tests	5](#_Toc207807157)

[Table S.5 Effect of FIT programmes on stool-based screening uptake among older (80+) and younger (30-49) age groups	6](#_Toc207807158)

[Table S.6 Effect of FIT programmes on placebo outcomes uptake, results from difference-in-differences regression models	6](#_Toc207807159)

[Appendix C: Robustness check	7](#_Toc207807160)

[Table S.7 Effect of FIT programmes on stool-based screening uptake by intervention country, results from difference-in-differences regression models	8](#_Toc207807161)

[Figure S.2 Effect of FIT programmes on stool-based screening across education levels, by intervention country, results from difference-in-differences regression models	9](#_Toc207807162)

# Appendix A: Supplementary Tables

# Table S.1 European Health Interview Survey data availability and classification of countries as intervention and comparison groups

|  | **EHIS-1** | **EHIS-2** | **EHIS-3** |
| --- | --- | --- | --- |
| **Intervention group** | | | |
| BE | X | X | X |
| DK |  | X | X |
| LU^a^ |  | X | X |
| NL |  | X | X |
| **Comparison group** | | | |
| AT | X^b^ | X | X |
| BG | X | X | X |
| CY | X | X | X |
| DE |  | X | X |
| EE | X^b^ | X | X |
| EL | X | X | X |
| ES | X | X | X |
| FI |  | X | X |
| HU | X | X | X |
| IS |  | X | X |
| IT |  | X | X |
| LT |  | X | X |
| LV | X | X | X |
| MT | X | X | X |
| NO |  | X | X |
| PL | X | X | X |
| PT |  | X | X |
| RO | X | X | X |
| SE |  | X | X |
| SK | X | X | X |
| **Excluded from the analysis** | | | |
| CZ | X | X | X |
| FR | X | X | X^c^ |
| HR |  | X | X |
| IE |  | X | X |
| RS |  |  | X |
| SI | X | X | X |
| UK |  | X |  |

^a^ Although Luxembourg implemented a pilot programme, we classified it as having a programme because FIT kits were mailed to all eligible individuals between 2014 and 2019, as documented in reports[26] and confirmed by programme stakeholders.

^b^ No data was collected on stool-based screening uptake.

^c^ Microdata was not released to researchers.

# Table S.2 Stool-based screening uptake by education level, before and after FIT programme implementation, in intervention and comparison groups

|  | | **Education level** | | | | | | **Chi-2 test** |
| --- | --- | --- | --- | --- | --- | --- | --- | --- |
|  |  | **Lower** | | **Middle** | | **Higher** | |  |
|  |  | *n* | % | *n* | % | *n* | % | *P value* |
| **Pre-intervention (EHIS-2)** | | | | | | | | |
| Comparison group | <2 years | 6659 | 20.1 | 8186 | 22.5 | 4363 | 24.9 | <0.001 |
|  | In lifetime | 10 747 | 32.5 | 14 172 | 38.9 | 7481 | 42.6 | <0.001 |
| Intervention group | <2 years | 454 | 19.1 | 641 | 22.8 | 588 | 24.5 | <0.001 |
|  | In lifetime | 733 | 30.9 | 994 | 35.4 | 959 | 39.9 | <0.001 |
| **Post-intervention (EHIS-3)** | | | | | | | | |
| Comparison group | <2 years | 8912 | 27.1 | 11 058 | 25.9 | 7288 | 31.2 | <0.001 |
|  | In lifetime | 13 654 | 41.5 | 18 187 | 42.6 | 12 097 | 51.7 | <0.001 |
| Intervention group | <2 years | 1290 | 52.8 | 2028 | 58.9 | 1886 | 54.3 | <0.001 |
|  | In lifetime | 1577 | 64.5 | 2456 | 71.3 | 2370 | 68.2 | <0.001 |

Source: Second and third waves of the European Health Interview Survey (EHIS-2 and EHIS-3).

# Table S.3 Effect of FIT programmes on stool-based screening uptake by education level, results from difference-in-differences regression models

|  | **In lifetime** | | | **In the past 2 years** | | |
| --- | --- | --- | --- | --- | --- | --- |
|  | b | 95% CI | *P value* | b | 95% CI | *P value* |
| Intervention (ref.: comparison group) | -0.476 | -0.558, -0.394 | <0.001 | -0.318 | -0.402, -0.235 | <0.001 |
| Post (ref.: pre-intervention period) | 0.050 | -0.005, 0.105 | 0.075 | 0.035 | -0.026, 0.097 | 0.240 |
| Education level (ref.: higher) |  |  |  |  |  |  |
| lower | -0.066 | -0.093, -0.040 | <0.001 | -0.042 | -0.061, -0.023 | <0.001 |
| middle | -0.006 | -0.022, 0.009 | 0.390 | -0.004 | -0.018, 0.010 | 0.570 |
| DiD | 0.246 | 0.095, 0.397 | 0.003 | 0.278 | 0.124, 0.433 | 0.001 |
| DiD x Education level (ref.: higher) |  |  |  |  |  |  |
| DiD x lower | 0.035 | -0.065, 0.135 | 0.480 | 0.017 | -0.069, 0.103 | 0.680 |
| DiD x middle | 0.089 | 0.033, 0.145 | 0.003 | 0.064 | 0.015, 0.112 | 0.010 |
| Intercept | 0.721 | 0.687, 0.755 | <0.001 | 0.491 | 0.458, 0.524 | <0.001 |
| *n* | 202 976 |  |  | 202 976 |  |  |
| R2 | 0.24 |  |  | 0.15 |  |  |

Notes: DiD (difference-in-differences) denotes the *Intervention x Post* term. Models were adjusted for age, sex and country fixed effects. Standard errors were clustered at the country level.

CI = confidence interval.

# Appendix B: Model assumptions

Our DiD model compares changes in screening uptake between countries that implemented mailed FIT programmes and those that did not. This approach accounts for both observed and unobserved time-invariant differences between intervention and comparison countries while controlling for shared temporal trends in screening uptake. Under this framework, if trends in screening uptake were parallel between intervention and comparison countries before the introduction of FIT programmes, and if programme implementation was not driven by pre-existing trends, any post-intervention differences in screening uptake between the two groups can justifiably be attributed to the FIT programmes. In sum, for causal effect identification, the DiD approach relies on the assumption that, in the absence of FIT programmes, differences between intervention and comparison groups would remain constant over time. In our study, we could only assess the parallel trends assumption for one intervention country (Belgium), as the only preceding EHIS wave (EHIS-1) covered fewer countries. Additionally, to assess the validity of our causal identification strategy and the robustness of our findings, we performed falsification and placebo tests across all intervention and comparison countries using data from EHIS-2 and EHIS-3.

# Assessment of the parallel trends assumption

Figure S.1 provides a visual assessment of the parallel trends assumption. This analysis was limited to one intervention country (Belgium) and 10 countries in the comparison group, as fewer countries participated in EHIS-1, and the other intervention countries (Denmark, Luxembourg and the Netherlands) did not take part in this survey wave. Moreover, Austria and Estonia did not collect information on stool-based screening uptake in EHIS-1. As Figure S.1 shows, in the pre-intervention period, trends in both lifetime and past-two-year screening uptake appeared parallel between Belgium and the comparison group of countries. Following the intervention, as expected, screening uptake in Belgium increased, diverging from the trend in the comparison group.

# Figure S.1 Parallel trends assumption in stool-based screening uptake

Note: EHIS = European Health Interview Survey.

In addition to the visual assessment of parallel trends, we conducted a regression-based test for pre-intervention trend similarity (Table S.4). We regressed screening outcomes (lifetime and past-two-year stool-based screening uptake) on time (survey year), the intervention variable (Belgium versus the comparison group of countries) and their interaction. The interaction term for Belgium x EHIS-2 (lifetime uptake: -0.022, p=0.380; past-two-year uptake: 0.017, p=0.300) was not significant, providing no evidence of differential pre-intervention trends between Belgium and the comparison group before the intervention, hence being consistent with the parallel trends assumption.

# Table S.4 Regression-based test of pre-intervention trends in stool-based screening uptake, Belgium (intervention) versus comparison group of countries

|  | **In lifetime** | | | **In the past 2 years** | | |
| --- | --- | --- | --- | --- | --- | --- |
|  | b | 95% CI | *P value* | b | 95% CI | *P value* |
| Belgium (ref: comparison group) | 0.208 | 0.148, 0.268 | <0.001 | 0.106 | 0.054, 0.157 | 0.001 |
| Survey year (ref.: EHIS-1) |  |  |  |  |  |  |
| EHIS-2 | 0.097 | 0.044, 0.149 | 0.002 | 0.048 | 0.014, 0.083 | 0.010 |
| EHIS-3 | 0.167 | 0.048, 0.286 | 0.011 | 0.105 | 0.002, 0.209 | 0.046 |
| Belgium x survey year (ref.: EHIS-1) |  |  |  |  |  |  |
| Belgium x EHIS-2 | -0.022 | -0.074, 0.031 | 0.380 | 0.017 | -0.017, 0.051 | 0.300 |
| Belgium x EHIS-3 | 0.096 | -0.022, 0.215 | 0.100 | 0.145 | 0.041, 0.248 | 0.011 |
| Intercept | -0.003 | -0.063, 0.058 | 0.930 | 0.006 | -0.045, 0.057 | 0.800 |
| R2 | 0.13 |  |  | 0.08 |  |  |
| *n* | 126 812 |  |  | 126 812 |  |  |

Note: the comparison group included 10 countries which participated in all three waves of the EHIS and collected information on stool-based screening uptake. Models were adjusted for country fixed effects. Standard errors were clustered at the country level.

CI = confidence interval.

# Falsification and placebo tests

We conducted falsification and placebo tests to further evaluate the validity of our difference-in-differences (DiD) approach. These tests help assess the robustness of the observed effects and detect whether they may be driven by alternative explanations, such as unmeasured confounding or spurious correlations.

First, we performed a falsification test by applying the DiD estimation to populations not eligible for the FIT programmes, i.e. not exposed to the intervention (Table S.5). Specifically, we analysed individuals aged 30-49 (n=149 419) and those aged 80 and above (n=27 990). The absence of an effect in these non-targeted groups supports the interpretation that the observed effects among the eligible population are attributable to the intervention itself, rather than to broader underlying trends. As shown in Table S.5, FIT programmes had no statistically significant effects on screening uptake among these age groups. These results indicate that the intervention may not have influenced screening uptake for individuals outside the target population (50-74 years old), providing further support to the validity of our counterfactual comparison.

# Table S.5 Effect of FIT programmes on stool-based screening uptake among older (80+) and younger (30-49) age groups

|  | **In lifetime** | | | **In the past 2 years** | | |
| --- | --- | --- | --- | --- | --- | --- |
|  | b | 95% CI | *P value* | b | 95% CI | *P value* |
| **80 years old and older** |  |  |  |  |  |  |
| Intervention (ref.: comparison group) | -0.530 | -0.571, -0.490 | <0.001 | -0.340 | -0.368, -0.312 | <0.001 |
| Post (ref.: pre-intervention period) | 0.048 | 0.005, 0.091 | 0.031 | 0.014 | -0.013, 0.041 | 0.300 |
| DiD | 0.025 | -0.037, 0.087 | 0.420 | 0.009 | -0.035, 0.053 | 0.680 |
| Intercept | 0.760 | 0.727, 0.793 | <0.001 | 0.453 | 0.432, 0.474 | <0.001 |
| R2 | 0.22 |  |  | 0.09 |  |  |
| *n* | 27 990 |  |  | 27 990 |  |  |
| **30 to 49 years old** |  |  |  |  |  |  |
| Intervention (ref.: comparison group) | -0.424 | -0.434, -0.415 | <0.001 | -0.228 | -0.237, -0.220 | <0.001 |
| Post (ref.: pre-intervention period) | 0.018 | 0.002, 0.034 | 0.033 | 0.014 | 0.003, 0.025 | 0.012 |
| DiD | -0.014 | -0.034, 0.007 | 0.190 | -0.017 | -0.035, 0.002 | 0.070 |
| Intercept | 0.528 | 0.521, 0.535 | <0.001 | 0.280 | 0.276, 0.285 | <0.001 |
| R2 | 0.12 |  |  | 0.06 |  |  |
| *n* | 149 419 |  |  | 149 419 |  |  |

Notes: DiD (difference-in-differences) denotes the *Intervention x Post* term. Models were adjusted for country fixed effects. Standard errors were clustered at the country level.

CI = confidence interval.

Second, we considered two placebo outcomes that should not be affected by FIT programmes: cholesterol and blood sugar checks (Table S.6). If the intervention were associated with changes in these outcomes, it would raise concerns about potential bias or misspecification in our DiD identification strategy, namely, whether the observed effects can truly be attributed to the intervention rather than to unmeasured confounding or broader temporal trends. Conversely, the absence of such effects would strengthen the credibility of our approach and suggest that the results are unlikely to be driven by broader changes in healthcare utilisation. As shown in Table S.6, we found no statistically significant effects of the FIT programmes on the placebo outcomes, providing additional support for our identification strategy.

# Table S.6 Effect of FIT programmes on placebo outcomes, results from difference-in-differences regression models

|  | **In lifetime** | | | **In the past year** | | |
| --- | --- | --- | --- | --- | --- | --- |
|  | b | 95% CI | *P value* | b | 95% CI | *P value* |
| **Cholesterol check** | | | | | | |
| Intervention (ref.: comparison group) | -0.118 | -0.123, -0.113 | <0.001 | -0.151 | -0.165, -0.136 | <0.001 |
| Post (ref.: pre-intervention period) | 0.010 | 0.003, 0.017 | 0.007 | 0.015 | -0.005, 0.036 | 0.130 |
| DiD | 0.000 | -0.010, 0.010 | 0.980 | -0.011 | -0.040, 0.017 | 0.420 |
| Intercept | 0.976 | 0.972, 0.979 | <0.001 | 0.704 | 0.694, 0.714 | <0.001 |
| R2 | 0.03 |  |  | 0.05 |  |  |
| *n* | 204 717 |  |  | 204 717 |  |  |
| **Blood sugar check** | | | | | | |
| Intervention (ref.: comparison group) | -0.137 | -0.142, -0.132 | <0.001 | -0.146 | -0.162, -0.130 | <0.001 |
| Post (ref.: pre-intervention period) | 0.010 | 0.002, 0.017 | 0.016 | 0.017 | -0.004, 0.038 | 0.110 |
| DiD | -0.001 | -0.011, 0.009 | 0.870 | -0.013 | -0.045, 0.019 | 0.400 |
| Intercept | 0.970 | 0.966, 0.974 | <0.001 | 0.702 | 0.692, 0.713 | <0.001 |
| R2 | 0.03 |  |  | 0.04 |  |  |
| *n* | 203 921 |  |  | 203 921 |  |  |

Notes: DiD (difference-in-differences) denotes the *Intervention x Post* term. Models were adjusted for country fixed effects. Standard errors were clustered at the country level.

CI = confidence interval.

# Appendix C: Robustness check

As a robustness check, we repeated the main analysis separately for the four intervention countries, comparing each of them to the comparison group of countries (Table S.7 and Figure S.2). This allowed us to assess the consistency of effects, considering the heterogeneity in programme implementation and national contexts. Notably, in some intervention countries (Denmark and the Netherlands), the launch of the FIT programme partially overlapped with the timing of the EHIS-2 data collection, and Luxembourg’s programme consisted of a single round of invitations.

# Table S.7 Effect of FIT programmes on stool-based screening uptake by intervention country, results from difference-in-differences regression models

|  | **Belgium** | | | **Denmark** | | | **Luxembourg** | | | **The Netherlands** | | |
| --- | --- | --- | --- | --- | --- | --- | --- | --- | --- | --- | --- | --- |
|  | b | 95% CI | *P value* | b | 95% CI | *P value* | b | 95% CI | *P value* | b | 95% CI | *P value* |
| **In lifetime** | | | | | | | | | | | | |
| Intervention (ref.: comparison group) | -0.486 | -0.509, -0.463 | <0.001 | -0.285 | -0.308, -0.262 | <0.001 | -0.272 | -0.295, -0.249 | <0.001 | -0.567 | -0.590, -0.545 | <0.001 |
| Post (ref.: pre-intervention period) | 0.059 | 0.013, 0.106 | 0.015 | 0.059 | 0.013, 0.106 | 0.015 | 0.059 | 0.013, 0.106 | 0.015 | 0.059 | 0.013, 0.106 | 0.015 |
| DiD | 0.128 | 0.082, 0.175 | <0.001 | 0.292 | 0.245, 0.338 | <0.001 | 0.114 | 0.067, 0.160 | <0.001 | 0.484 | 0.437, 0.530 | <0.001 |
| Intercept | 0.767 | 0.744, 0.790 | <0.001 | 0.767 | 0.744, 0.790 | <0.001 | 0.767 | 0.744, 0.790 | <0.001 | 0.767 | 0.744, 0.790 | <0.001 |
| R2 | 0.23 |  |  | 0.24 |  |  | 0.23 |  |  | 0.24 |  |  |
| **In the past two years** | | | | | | | | | | | | |
| Intervention (ref.: comparison group) | -0.338 | -0.359, -0.317 | <0.001 | -0.175 | -0.196, -0.154 | <0.001 | -0.259 | -0.280, -0.238 | <0.001 | -0.413 | -0.434, -0.392 | <0.001 |
| Post (ref.: pre-intervention period) | 0.044 | 0.002, 0.087 | 0.043 | 0.044 | 0.002, 0.087 | 0.043 | 0.044 | 0.002, 0.087 | 0.043 | 0.044 | 0.002, 0.087 | 0.043 |
| DiD | 0.140 | 0.098, 0.183 | <0.001 | 0.293 | 0.250, 0.336 | <0.001 | 0.162 | 0.119, 0.205 | <0.001 | 0.514 | 0.471, 0.557 | <0.001 |
| Intercept | 0.515 | 0.494, 0.536 | <0.001 | 0.515 | 0.494, 0.536 | <0.001 | 0.515 | 0.494, 0.536 | <0.001 | 0.515 | 0.494, 0.536 | <0.001 |
| R2 | 0.14 |  |  | 0.15 |  |  | 0.14 |  |  | 0.15 |  |  |
| *n* | 190 377 |  |  | 191 684 |  |  | 188 195 |  |  | 190 822 |  |  |

Notes: DiD (difference-in-differences) denotes the *Intervention x Post* term. Models were estimated separately for each intervention country, adjusting for country fixed effects. Standard errors were clustered at the country level.

CI = confidence interval.

# Figure S.2 Effect of FIT programmes on stool-based screening across education levels, by intervention country, results from difference-in-differences regression models

Notes: Bars indicate 95% confidence intervals. DiD (difference-in-differences) denotes the *Intervention x Post* term. It corresponds to the effect of FIT programmes on screening uptake among the higher education group, i.e. the reference category in the three-way interaction between *Intervention x Post x Education*. Models were adjusted for age, sex and country fixed effects. Standard errors were clustered at the country level.
